# Robot-assisted laparoscopic nephrectomy: early outcome measures with the implementation of multimodal analgesia and intrathecal morphine via the acute pain service

**DOI:** 10.1007/s00345-024-04801-z

**Published:** 2024-03-04

**Authors:** Minhthy N. Meineke, Matthew V. Losli, Jacklynn F. Sztain, Matthew W. Swisher, Wendy B. Abramson, Erin I. Martin, Timothy J. Furnish, Amirali Salmasi, Ithaar H. Derweesh, Rodney A. Gabriel, Engy T. Said

**Affiliations:** 1 Division of Acute Pain, Department of Anesthesiology, University of California, 9400 Campus Point Dr, San Diego, La Jolla, CA 92037 USA; 2 Department of Anesthesiology, University of California, San Diego, La Jolla, CA USA; 3 Division of Obstetric Anesthesia, Department of Anesthesiology, University of California, San Diego, La Jolla, CA USA; 4 Division of Pain, Department of Anesthesiology, University of California, San Diego, La Jolla, CA USA; 5 Department of Urology, University of California, San Diego, La Jolla, CA USA

**Keywords:** Acute pain service, Robotic nephrectomy, Intrathecal morphine, Multimodal analgesia

## Abstract

**Purpose:**

The objective of this study was to perform a retrospective cohort analysis, in which we measured the association of an acute pain service (APS)-driven multimodal analgesia protocol that included preoperative intrathecal morphine (ITM) compared to historic controls (i.e., surgeon-driven analgesia protocol without ITM) with postoperative opioid use.

**Methods:**

This was a retrospective cohort study in which the primary objective was to determine whether there was a decrease in median 24-h opioid consumption (intravenous morphine equivalents [MEQ]) among robotic nephrectomy patients whose pain was managed by the surgical team prior to the APS, versus pain managed by APS. Secondary outcomes included opioid consumption during the 24–48 h and 48–72 h period and hospital length of stay. To create matched cohorts, we performed 1:1 (APS:non-APS) propensity score matching. Due to the cohorts occurring at the different time periods, we performed a segmented regression analysis of an interrupted time series.

**Results:**

There were 76 patients in the propensity-matched cohorts, in which 38 (50.0%) were in the APS cohort. The median difference in 24-h opioid consumption in the pre-APS versus APS cohort was 23.0 mg [95% CI 15.0, 31.0] (*p* < 0.0001), in favor of APS. There were no differences in the secondary outcomes. On segmented regression, there was a statistically significant drop in 24-h opioid consumption in the APS cohort versus pre-APS cohort (*p* = 0.005).

**Conclusions:**

The implementation of an APS-driven multimodal analgesia protocol with ITM demonstrated a beneficial association with postoperative 24-h opioid consumption following robot-assisted nephrectomy.

## Introduction

Robot-assisted partial (RAPN) and radical nephrectomy (RARN) have become widely accepted surgical approaches for resecting renal tumors in the clinical setting. In addition to incisional pain, patients can experience peritoneal pain secondary to carbon dioxide insufflation, visceral pain, and referred posterior shoulder pain. For these reasons, acute postoperative pain management of these patients can be challenging. Visceral pain comprises most pain experienced within the first 24 h, which generally decreases on subsequent postoperative days (POD) [1]. Local anesthetics administered into the intraperitoneum may help address incisional pain, but have little impact on visceral pain [1].

There have been limited studies done to evaluate specific analgesic therapies for RAPN and RARN. Intrathecal morphine (ITM) is known to provide significant analgesia for approximately 24 h at a much lower dose compared to systemic opioids and has the advantage of not requiring a catheter placement for continuous infusions [2]. However, it does have an increased risk for respiratory depression, especially when patients concurrently receive systemic opioids [3, 4]. Due to this risk, it has been suggested that neuraxial opioids may only be beneficial in laparoscopic urologic patients if there is a high probability of converting to an open procedure [4, 5]. It has been described that ITM in patients undergoing robot-assisted radical prostatectomy has reduced postoperative pain, as well as reduced opioid use, on POD1 compared to the control group [5]. ITM has also been shown to reduce postoperative pain, total hospital systemic opioid consumption, and length of hospital stay in laparoscopic bariatric surgery patients [6]. However, there is a paucity of literature looking at the effect of ITM and a dedicated acute pain service (APS) on the postoperative pain of RAPN and RARN patients.

The objective of this study was to perform a retrospective cohort analysis, in which we measured the association of an APS-driven multimodal analgesia protocol that included preoperative ITM compared to historic controls (i.e., surgeon-driven analgesia protocol without ITM) with postoperative opioid use. We hypothesized that the implementation of a multimodal analgesia protocol with ITM would be associated with decreased postoperative opioid use.

## Methods

### Study population

The resulting dataset remained de-identified and did not contain sensitive patient-health information as defined by the institutional Human Research Protections Program, and, therefore, was exempt from the informed consent requirement and approved by our institutional review board. Data were collected retrospectively from the data warehouse of our institution. All data for surgical patients that were scheduled for a robot-assisted partial nephrectomy from 2020 to 2021 were extracted.

This was a retrospective cohort study in which the primary objective was to determine whether there was a decrease in median 24-h opioid consumption (intravenous morphine equivalents [MEQ]) among robotic nephrectomy patients whose pain was managed by the surgical team prior to the APS, versus pain managed by APS. Secondary outcomes included opioid consumption during the 24–48 h and 48–72 h period and length of hospital stay. APS screened patients scheduled for RAPN and RARN for candidacy of ITM. Patients who were on anticoagulation, coagulopathic, allergic to morphine, and refused ITM injection were excluded from the study. ITM injection was performed in the preoperative holding area prior to the patient being transported to the OR suite. All APS patients received perioperative multimodal analgesic regimen. Preoperatively, patients received 975 mg PO acetaminophen (APAP). Based on age and preoperative renal function, patients received 200–300 mcg ITM. All patients had their surgeries performed by one of two urologic oncology surgeons. Insufflation pressures of 15 mmHg were used intraoperatively. Postoperatively, patients’ pain was managed primarily by APS. Patients were prescribed scheduled PO APAP and as needed IV opioids for breakthrough pain. Based on preoperative renal function, majority of postoperative patients also received scheduled low-dose ketorolac (15 mg IV) for 24 h. On postoperative day 1, patients were transitioned from prn IV opioids to PO opioids. PO opioid type and dose were tailored to each patient based on IV opioid requirement, whether or not the patient was opioid naïve and their age. Discharge opioid prescription recommendations were also provided to the surgeons. Prior to APS involvement, surgeons inconsistently prescribed scheduled APAP and rarely utilized ketorolac. Postoperatively, patients received a standard prn IV opioid set (0.5–1 mg hydromorphone every 4 h prn) or prn PO opioid order set (oxycodone 5–10 mg PO every 6 h prn with morphine 2 mg IV prn breakthrough pain). Most patients also received a discharge prescription for oxycodone 5 mg PO #20 without consideration of inpatient opioid requirement. Other data collected included patient age, sex, American Society of Anesthesiologists (ASA) classification score, body mass index (BMI), and history of preoperative opioid use (defined as patients prescribed and confirmed use of preoperative opioids).

### Statistical analysis

All statistical analyses were performed using R (Version 4.2.2). To compare the primary and secondary outcomes in the unmatched cohorts, we used the Wilcoxon rank sum test. The median difference and 95% confidence interval (CI) were calculated using the Hodges–Lehman estimator. To create matched cohorts, we performed 1:1 (APS:non-APS) propensity score matching using nearest neighbor-matching without replacement. For this, we set the caliper at 0.2 standard deviations of the logit of the estimated propensity score. The propensity score for each cohort was calculated using logistic regression based on BMI, age, preoperative opioid use, sex, and ASA score. The covariates were included due to their theoretical association with postoperative pain. An absolute standardized mean difference less than or equal to 0.2 for each covariate was considered adequate for balanced matching. To compare the primary and secondary outcomes in the matched cohorts, we used the Wilcoxon signed rank test. A *p* < 0.05 was considered statistically significant.

Due to the cohorts occurring at the different time periods, we performed a segmented regression analysis of an interrupted time series to model trends in the primary outcome during the: (1) pre-APS study period; (2) immediately after APS (multimodal analgesia with ITM) was initiated; and (3) APS study period. To perform a segmented regression, we utilized the following regression equation:

$$Y = b0 + b1T + b2D + b3P + e$$

where (*Y*) = the outcome variable (24-h postoperative opioid consumption measured in MEQs); (*T*) = continuous variable which indicates time passed from the start of the observation period; (*D*) = a variable indicating observation collected before or after initiation of APS; and (*P*) = a continuous variable indicating time passed since APS was implemented in this surgical population. Statistically significant estimates for *T* would indicate a trend change in the outcome during the pre-APS period; for *D* would indicate an immediate change in the outcome when APS was started; and for *P* would indicate a trend change in the outcome during the time period from start of APS to end of study period.

## Results

**Fig. 1:**
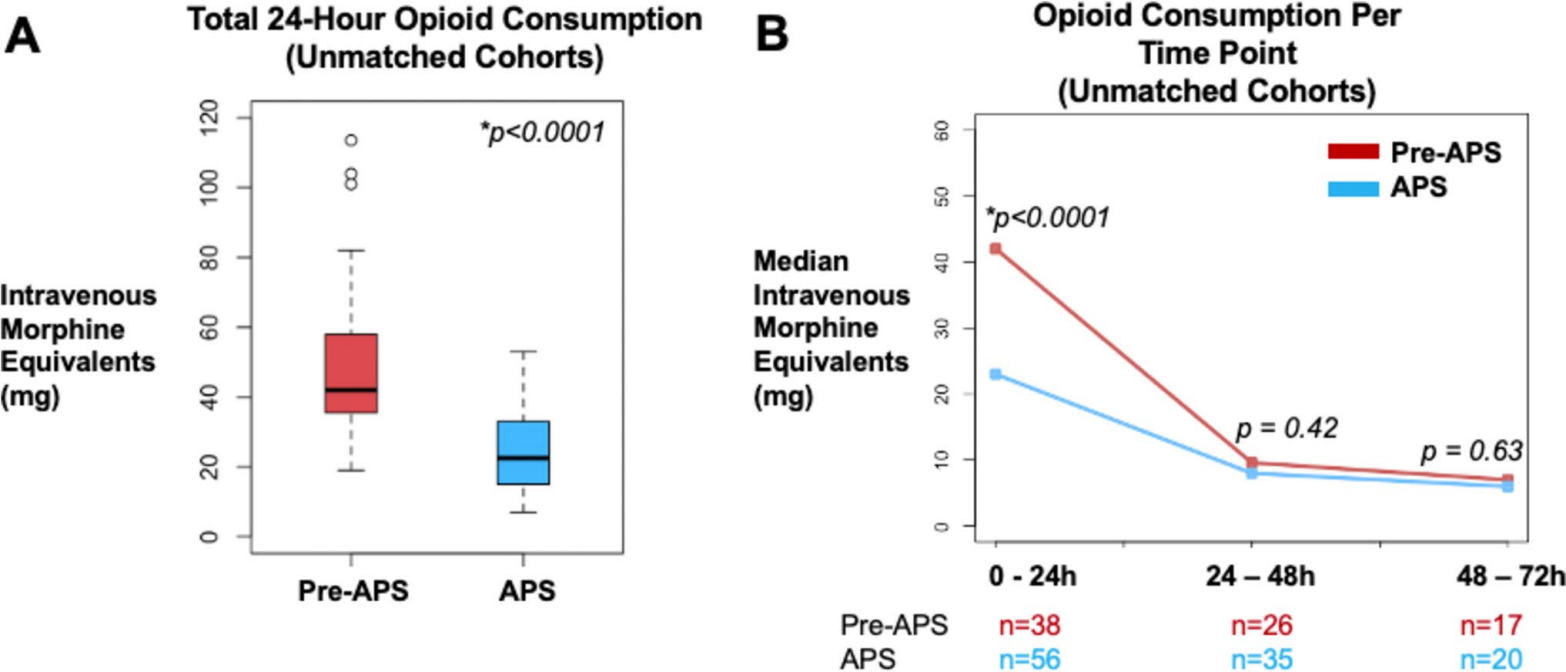
Analysis of the unmatched cohorts. **A** Box plot illustrating the difference in the median 24-h opioid consumption between the pre-APS and APS cohorts; **B** Line plot illustrating the difference in the median opioid consumption at multiple time points. Abbreviations: APS, acute pain service

There were a total of 94 patients included in the analysis, in which 56 (60.0%) were in the APS cohort (Table 1). The median [quartiles] 24-h opioid consumption in the pre-APS versus APS cohort was 42.0 mg [35.9, 58.0] versus 22.5 mg [15.0, 33.0], with a median difference of 19.5 mg [95% CI 11.0, 29.0] (*p* < 0.0001) (Fig. 1A). There was no difference in median opioid consumption at the 24–48 h and 48–72 h time periods (Fig. 1B), and no difference in length of hospital stay in the pre-APS versus APS cohorts (2 days [1.4, 3.0] versus 2 days [1.3, 3.0], respectively, *p* = 0.55). Furthermore, there was no difference in the amount of outpatient opioids prescribed at discharge in the pre-APS versus APS cohorts (16 mg [10, 23] versus 20 mg [0, 24.5], respectively, *p* = 0.85).

**Table 1:** Patient characteristics of the pre-APS and APS cohorts in unmatched and propensity-matched cohorts

	Unmatched cohorts		Propensity-matched cohorts		SMD
	Pre-APS	APS	Pre-APS	APS	
Total	38	56	38	38	–
Age (years), mean [SD]	63.1 [13.3]	58.9 [14.3]	63.1 [13.3]	61.2 [14.4]	0.09
BMI (kg/m2), mean [SD]	30.0 [6.8]	28.6 [7.6]	30.0 [6.8]	29.1 [7.1]	0.08
ASA score, mean [SD]	2.8 [0.4]	2.7 [0.5]	2.8 [0.4]	2.8 [0.3]	0.08
Male sex, *n* [%]	17 [44.7]	35 [64.3]	17 [44.7]	20 [52.6]	0.18
Preoperative opioid use History, *n* [%]	5 [13.2]	9 [16.0]	5 [13.2]	8 [21.1]	0.19

*APS* acute pain service; *ASA* American Society of Anesthesiologists; *BMI* body mass index; *SD* standard deviation; *SMD* absolute standardized mean difference

**Fig. 2:**
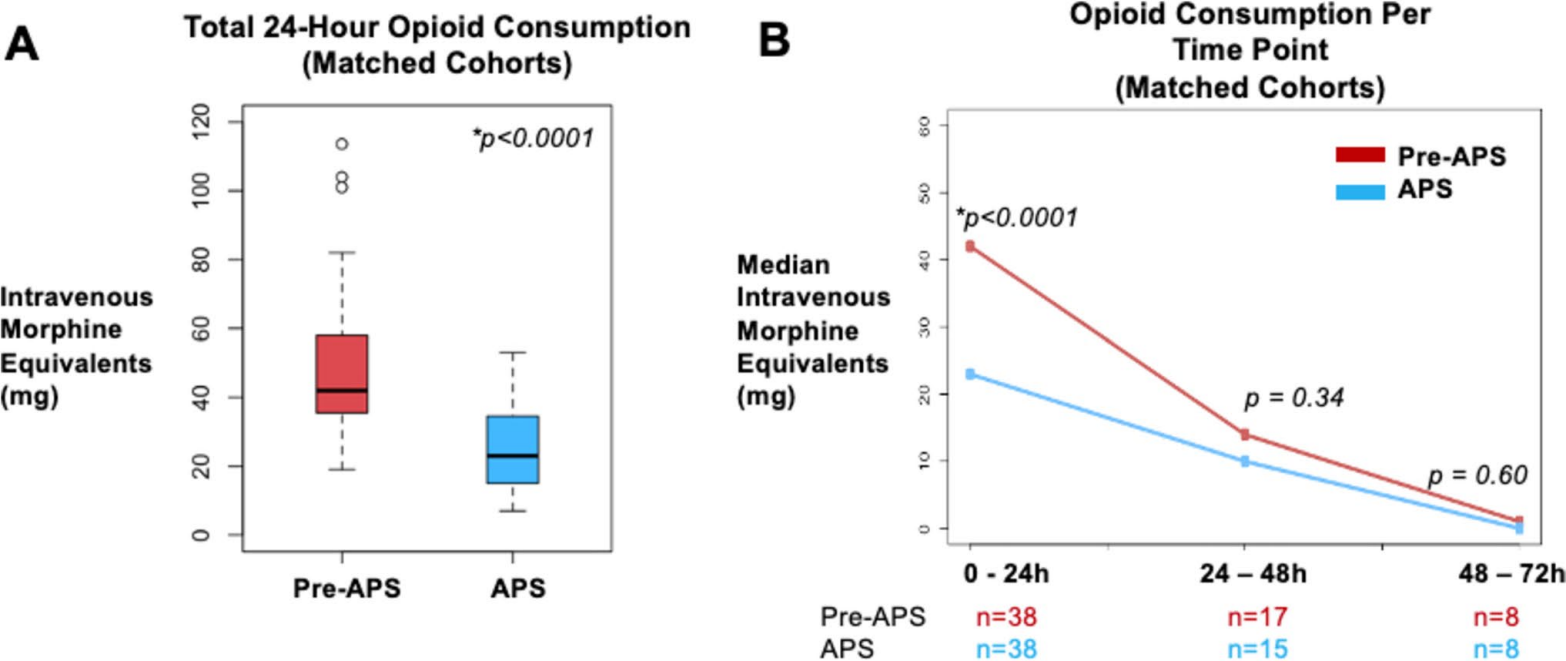
Analysis of the propensity-matched cohorts. **A** Box plot illustrating the difference in the median 24-h opioid consumption between the pre-APS and APS cohorts; **B** Line plot illustrating the difference in the median opioid consumption at multiple time points. Abbreviations: APS, acute pain service

We created propensity-matched cohorts controlling for age, sex, preoperative opioid use, body mass index, and ASA score. There were 76 patients in this analysis, in which 38 (50.0%) were in the APS cohort (Table 1). The absolute standardized mean difference between each confounder was less than 0.2. The median [quartiles] 24-h opioid consumption in the pre-APS versus APS cohort was 42.0 mg [35.9, 58.0] versus 23.0 mg [15.0, 34.5], with a median difference of 23.0 mg [95% CI 15.0, 31.0] (*p* < 0.0001) (Fig. 2A). There was no difference in median opioid consumption at the 24–48 h and 48–72 h time periods (Fig. 2B) and no difference in length of hospital stay in the pre-APS versus APS cohorts (2 days [1.4, 3.0] versus 2 days [1.4, 3.0], respectively, *p* = 0.92). Furthermore, there was no difference in the amount of outpatient opioids prescribed at discharge in the pre-APS versus APS cohorts (16 mg [10, 23] versus 20 mg [10.5, 27.5], respectively, *p* = 0.57).

**Fig. 3:**
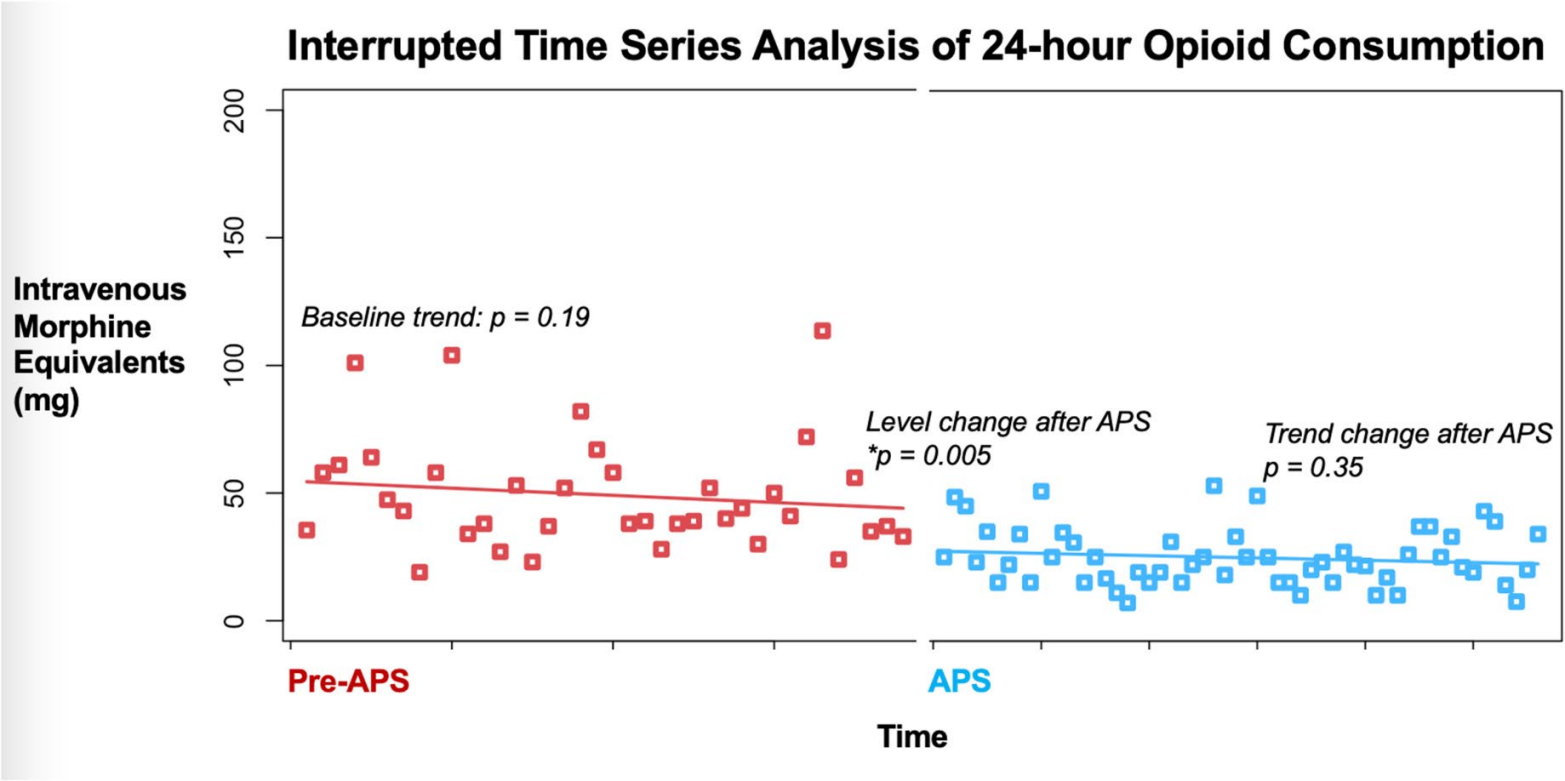
Illustration plotting an interrupted time series analysis of 24-h opioid consumption during the pre-APS and APS time period. Abbreviations: APS, acute pain service

We subsequently performed a segmented regression analysis using the entire dataset and controlled for age, sex, preoperative opioid use, body mass index, and ASA score (Fig. 3). There was no statistically significant trend in changes of 24-h opioid use during the pre-APS interval (*p* = 0.19). Furthermore, there was no statistically significant trend in 24-h opioid consumption during the APS interval (*p* = 0.35). However, there was a statistically significant drop in 24-h opioid consumption in the APS cohort versus pre-APS cohort (*p* = 0.005).

## Discussion

In this retrospective cohort study, we demonstrated that the implementation of an APS-driven multimodal analgesia protocol with preoperative ITM was associated with an approximate 50% reduction in 24-h opioid consumption following robot-assisted nephrectomy. Based on our interrupted time series analysis, we demonstrated that immediately after integrating APS into the pain management plan for these patients, there was a significant decrease in opioid consumption. This effect was furthermore sustained throughout the time period after the involvement of APS was established for this surgical population.

There is a paucity of literature regarding specific techniques to reduce postoperative pain in RAPN and RARN patients. There was a recent study that found that ultrasound-guided transversus abdominis plane block reduced postoperative opioid use and somatic pain in RAPN patients, but it did not reduce visceral pain [7]. Both Shim et al. and Koning et al. demonstrated that ITM and spinal bupivacaine can reduce total systemic opioid consumption in robot-assisted laparoscopic prostatectomy patients [8, 9]. Similarly, Talwar et al. were able to eliminate the need for discharge opioid prescription in robot-assisted radical prostatectomy patients with a nonopioid analgesic pathway. However, the majority of RARN patients still required a discharge opioid prescription, which was consistent with our patient population [10]. This may reflect the visceral pain that is specific to RAPN and RARN patients. Our results are consistent with those of prior studies evaluating the impact of ITM on pain and systemic opioid use in other laparoscopic surgeries that reflect decreased systemic opioid use and pain within the first 24 h postoperatively [5, 6].

As Alexander’s review article described, pain after laparoscopy experienced after POD 2 was due to peritoneal inflammation or presence of gas which can persist for at least 3 days and was best managed with a combination of non-steroidal anti-inflammatory drugs and systemic opioids [1]. Our APS sought to manage both somatic and visceral pain with preoperative ITM injection. The results are promising with regard to having a potential impact on total hospital-stay opioid use, without any complications typically associated with ITM (respiratory depression and pruritus). The findings within our cohort analysis were consistent with prior ITM dose-dependent studies that demonstrated no increased risk for respiratory depression when conservative ITM dosing (< 400 mcg) was used [3, 4]. In addition to a conservative ITM dosing, this is most likely secondary to having a dedicated APS that managed patients’ postoperative systemic opioids instead of traditionally having a surgical service whose limited knowledge of concurrent ITM and systemic opioid management would suggest a higher risk for opioid overdose. Our study did not see a statistically significant impact on outpatient opioid prescription. However, our study was underpowered for this outcome.

Pain secondary to laparoscopic surgery cannot only be addressed by local anesthetic infiltration, non-steroidal anti-inflammatory medication, and systemic opioid due to the complexity of pain based on the location of the surgery and its duration [11]. Future iterations of our protocol need to include postoperative opioid discharge and need for outpatient opioid refill to evaluate the long-term impact on RARN and RAPN patients.

There are several limitations to this study–mainly due to the retrospective nature of the study design. With retrospective studies, there may be inherent biases that we would be unable to account for unless a subsequent prospective clinical trial was performed. For example, there may be information bias and confounding. In regard to information bias, the data used for this study were dependent on the accuracy of record collection based in the electronic medical record system. Furthermore, there may be several confounders that we were unable to control within our analyses. Another limitation is related to study power–in that, we did not detect differences in our secondary outcomes. This may be due to inadequate sample size and thus, future studies would need to ensure appropriate power to address those specific outcomes.

In conclusion, the implementation of an APS-driven multimodal analgesia protocol with ITM demonstrated a beneficial association with postoperative 24-h opioid consumption following robot-assisted nephrectomy. While no differences were found in opioid consumption for subsequent time points nor hospital length of stay, future studies would need to address these outcomes with larger sample sizes.

## Data Availability

The de-identified data may be shared via a data use agreement with the institution.
